# Genome-Wide Survey of the Potential Function of CrLBDs in *Catharanthus roseus* MIA Biosynthesis

**DOI:** 10.3390/genes15091140

**Published:** 2024-08-29

**Authors:** Chunhao Chang, Bingrun Yang, Xiaorui Guo, Chunyan Gao, Biying Wang, Xiaoju Zhao, Zhonghua Tang

**Affiliations:** 1 College of Chemistry, Chemical Engineering and Resource Utilization, Northeast Forestry University, Harbin 150040, China; doc_cch@nefu.edu.cn (C.C.); xruiguo@nefu.edu.cn (X.G.); gcy1269679217@163.com (C.G.); 2 Key Laboratory of Plant Ecology, Northeast Forestry University, Harbin 150040, China; 3 School of Biological Engineering, Dalian Polytechnic University, Dalian 116034, China; yangbr185855@163.com (B.Y.); biying_wang@outlook.com (B.W.); 4 Bioengineering Institute, Daqing Normal University, Daqing 163712, China

**Keywords:** LBD gene family, MIA biosynthesis, co-expression analysis, Y1H assays, *Catharanthus roseus*

## Abstract

*Catharanthus roseus* (*C*. *roseus*) can produce over 150 types of monoterpenoid indole alkaloids (MIAs), including vinblastine and vincristine, which are currently the primary sources of these alkaloids. Exploring the complex regulatory mechanisms of *C*. *roseus* is significant for resolving MIA biosynthesis. The Lateral Organ Boundaries Domain (LBD) is a plant-specific transcription factor family that plays crucial roles in the physiological processes of plant growth, stress tolerance, and specialized metabolism. However, the LBD gene family has not been extensively characterized in *C*. *roseus*, and whether its members are involved in MIA biosynthesis is still being determined. A total of 34 *C*. *roseus LBD* (*CrLBD*) genes were identified. RNA-Seq data were investigated to examine the expression patterns of *CrLBD* genes in various tissues and methyl jasmonate (MeJA) treatments. The results revealed that the Class Ia member *CrLBD4* is positively correlated with iridoid biosynthetic genes (*p* < 0.05, r ≥ 0.8); the Class IIb member CrLBD11 is negatively correlated with iridoid biosynthetic genes (*p* < 0.05, r ≤ −0.8). Further validation in leaves at different growth stages of *C. roseus* showed that *CrLBD4* and *CrLBD11* exhibited different potential expression trends with iridoid biosynthetic genes and the accumulation of vindoline and catharanthine. Yeast one-hybrid (Y1H) and subcellular localization assays demonstrated that CrLBD4 and CrLBD11 could bind to the “aattatTCCGGccgc” *cis*-element and localize to the nucleus. These findings suggest that CrLBD4 and CrLBD11 may be potential candidates for regulating MIA biosynthesis in *C. roseus*. In this study, we systematically analyzed the CrLBD gene family and provided insights into the roles of certain CrLBDs in the MIA biosynthesis of *C. roseus*.

## 1. Introduction

In medicinal plant research, identifying the genes involved in plant metabolic pathways and elucidating their regulatory mechanisms is an important research direction [1,2,3]. With the advancement of high-throughput sequencing technologies, we now have access to an extensive array of genetic data. These data and continuously improving bioinformatics tools significantly enhance our ability to discover new genes [4,5,6,7]. Gene discovery typically relies on analyzing the association between gene expression and specific metabolic molecules, and gene family analysis has been widely applied as a method for systematically identifying specific gene families within a species [7]. Coupled with transcriptome analysis, gene family analysis has become an effective strategy for investigating the functions of candidate genes [8,9]. Thus, the abundant datasets support studying complex metabolic networks in medicinal plants and facilitate elucidating potential regulatory mechanisms through systematic analysis.

Plant gene families participate in complex regulatory networks involved in plant growth, development, stress tolerance, and specialized metabolism [10,11,12,13]. Among these, the plant-specific Lateral Organ Boundaries Domain (LBD) gene family is pivotal in forming lateral organ boundaries. These genes are involved in nearly all stages of plant development, including the development of embryos, roots, leaves, and inflorescences [14,15,16,17,18]. The Lateral Organ Boundaries (LOBs) were first identified in *Arabidopsis thaliana* (*A. thaliana*). The 43 LBD gene family members can be divided into two classes, Class I (Ia, Ib, Ic, Id, and Ie) and Class II (IIa and IIb), according to the structure of the LOB domain [19]. Class I members have a fully conserved CX2CX6CX3C zinc finger-like motif at the N-terminus with potential DNA-binding capabilities. Their GAS (Gly-Ala-Ser) block, located in the middle of the LOB structural domain, has conserved proline residues that play a crucial role in the biological function of LBD proteins in *A. thaliana* [20]. The leucine zipper-like structure (LX6LX3LX6L) allows the formation of coiled-coil protein dimerization [19]. By contrast, Class II LBD proteins only contain a conserved CX2CX6CX3C zinc finger-like motif. However, studies have shown that proteins like ZmLBD5 and ZmLBD33 in maize can form homo- and hetero-dimers, similar to Class I members, even without the leucine zipper-like coiled-coil domain. This suggests that alternative motifs or interactions could facilitate dimerization in Class II LBD proteins [21]. Functional analyses in *A. thaliana* and other higher plants have shown that Class I members are mainly involved in developing early lateral organs, including lateral vegetative and floral organs. Class II members might be involved in metabolic processes such as anthocyanin synthesis, affecting nitrogen responses [9,22].

The LBD gene family is primarily recognized for its role in mediating growth and developmental processes in plants, exemplified by the function of LBD16 in initiating lateral root development in *A. thaliana* [23]. Recent research has expanded the understanding of the LBD family, revealing that Class II LBD transcription factors specifically regulate plant-specialized metabolisms. Notably, Class IIb members, including LBD37, LBD38, and LBD39 in *A. thaliana*, have been identified as regulators of anthocyanin biosynthesis pathways [22]. In *Salvia miltiorrhiza* (*S*. *miltiorrhiza*), SmLBD50 has been found to interact with a network of transcription factors—SMeJAAZ1, SmMYB36/37, SmbHLH37, and SmMYC2a/2b—to inhibit the biosynthesis of phenolic compounds and anthocyanins [24]. Moreover, the scope of functional roles of Class I LBD members extends beyond the modulation of lateral organ development. For instance, AbLBD1 promotes root development and concurrently reduces alkaloid synthesis in *Atropa belladonna* (*A. belladonna*), indicating a dual regulatory capability that impacts specialized metabolite pathways [25]. Collectively, these findings propose that LBD transcription factors, across both classes, potentially exert a substantial influence on the regulation of specialized metabolism, advocating for a re-evaluation of their roles within plant biology.

To date, the LBD gene family has been systematically investigated for some plants, such as *A. thaliana*, *Oryza sativa*, *Malus domestica*, and *S*. *miltiorrhiza* [26,27,28]. The critical medicinal plant *C. roseus* is estimated to have more than 150 monoterpenoid indole alkaloids (MIAs), among which vinblastine and vincristine are crucial precursor compounds for clinical anticancer drugs [29]. However, their low yield fails to meet market demand. Therefore, understanding the biosynthesis and regulatory mechanisms of MIAs in *C. roseus* and deciphering the complex regulatory networks involved in MIA biosynthesis is critical for increasing their yield. While the complete metabolic pathway of MIAs in *C. roseus* has been largely elucidated, the influence of transcriptional regulation, post-transcriptional regulation, and protein modifications on MIA biosynthesis requires further research. Transcription factors such as CrORCA3, CrMYC2, and CrWRKY1 play crucial roles in the specialized metabolism of *C. roseus* [30,31,32]. Changes in their expression levels can significantly affect the accumulation of MIAs. Thus, exploring the roles of additional transcription factors in the MIA biosynthesis of *C. roseus* is of profound significance for enhancing MIA accumulation and meeting market demand. However, the LBD gene family has not been extensively characterized in *C. roseus*, and whether its members are involved in MIA biosynthesis is unknown. Exploring the biological functions of LBD gene family members can enrich the regulatory network of MIA biosynthesis in *C. roseus*.

In this study, we systematically identified the LBD gene family in *C. roseus* and used public RNA-Seq data to analyze the co-expression of *CrLBD4* and *CrLBD11* with iridoid biosynthesis genes. By integrating RT-qPCR data and HPLC analysis from different leaf ages of *C. roseus*, we found that *CrLBD4* exhibits a positive correlation with iridoid pathway metabolic genes and specialized metabolites, while *CrLBD11* shows a negative correlation. Subcellular localization and Y1H assays preliminarily suggest that CrLBD4 and CrLBD11 may be transcription factors involved in specialized metabolites in *C. roseus*. These findings may provide critical genetic elements and theoretical foundations for studying the regulatory network mechanisms of the specialized metabolism in *C. roseus* and offer insights for future breeding programs.

## 2. Materials and Methods

### 2.1. Identification of LBD Gene Family in C. roseus

To find the candidate LBD gene family members in the *C. roseus* genome, the whole genome, annotation files, and protein sequences of version 3 were downloaded from the DRYAD database (https://doi.org/10.5061/dryad.d2547d851, accessed on 18 February 2024). The LBD protein sequences of *A. thaliana* were retrieved from the TAIR database (https://www.arabidopsis.org/, accessed on 18 February 2024) [33]. Local BLASTp 2.10.0 (http://blast.ncbi.nlm.nih.gov, accessed on 26 November 2023) tools with E-value < 1 × 10^−5^, available from the National Center for Biotechnology Information (NCBI) website, were used to search the genome sequence of CrLBD with the known AtLBD sequences as the query [34]. The LOB files (PF03195) obtained from the Pfam database (http://pfam.xfam.org/, accessed on 20 February 2024) were used as the query for the Hidden Markov Model (HMM) search using the HMMER_v3.2.1 program with a pre-defined threshold of E-value < 1 × 10^−5^ [35]. To further confirm the CrLBD proteins, the data were continually verified by the Conserved Domain Database (CDD, https://www.ncbi.nlm.nih.gov/Structure/cdd/cdd.shtml, accessed on 20 February 2024) and Simple Modular Architecture Research Tool (SMART tool, http://smart.embl.de/, accessed on 20 February 2024). Finally, the acquired protein sequences of *C. roseus* were submitted to ExPASy (https://web.expasy.org, accessed on 20 February 2024) to calculate the physicochemical parameters like molecular weight (MW) and theoretical isoelectric points (pI) [36]. Chromosome localization was drawn using MapChart2.2 software [37]. Subcellular localization of CrLBD proteins was predicted using Cell-PLoc-2 (http://www.csbio.sjtu.edu.cn/bioinf/Cell-PLoc-2/, accessed on 20 February 2024) with default settings [38].

### 2.2. Multiple Sequence Alignment and Phylogenetic Analysis

Multiple sequence alignment of all CrLBD full-length protein sequences was performed using Muscle v3.8.1551 software. The unrooted phylogenetic tree was constructed using the ML method of IQ-TREE with 1000 bootstraps based on the WAG + F + R4 model. Additionally, the phylogenetic relationships of CrLBD proteins along with *A. thaliana* LBD proteins were also constructed using IQ-TREE based on the JTTDCMut + F + R8 model. The iTOL was used to visualize the phylogenetic tree.

### 2.3. Gene Structures, Conserved Motifs Analysis, and Cis-Element Analysis

The exon–intron structural information for *CrLBD* genes was obtained from the *C. roseus* gff3 file and displayed using the online software GSDS2.0 (http://gsds.gao-lab.org/, accessed on 21 February 2024) [39]. The MEME 5.5.3 was employed (https://meme-suite.org/meme/tools/meme, accessed on 21 February 2024) to identify the conserved motif of CrLBD proteins [40]. The parameters were employed as the following descriptions: repetitions, any number; maximum number of motifs, 10; and optimum width of each motif, 6–50 residues. The promoter sequences, 2000 bp upstream of the translational start site, of *CrLBD* genes were obtained from the *C. roseus* genome. Afterward, the online software PlantCARE (http://bioinformatics.psb.ugent.be/webtools/plantcare/html/, accessed on 25 February 2024) was employed to investigate putative *cis*-elements in the promoter region of *CrLBD* genes.

### 2.4. Chromosomal Distribution and Gene Duplication of CrLBD Genes

*CrLBD* genes were mapped to the eight chromosomes of *C. roseus* according to genome annotation information (gff3 files), and the results were visualized with Mapchart 2.32 software. The Multiple Collinearity Scan toolkit (MCScanX) and BLASTp were used to determine *CrLBD* gene synteny and collinearity. Then, Circos v 0.69-8 software was applied to express the syntenic relationship of the duplicated genes. To demonstrate the synteny of orthologous *LBD* genes obtained from *C. roseus* and other selected species (*A. thaliana*, *C. acuminata*, and *Ophiorrhiza pumila* (*O. pumila*)), we analyzed gene duplication events using the jcvi and visualized results.

### 2.5. Transcription Profiling Based on RNA-Seq Data and RT-qPCR Validation

The RNA-Seq datasets (under accession numbers PRJNA358259 and SRP005953) were downloaded from the Sequence Read Archive (SRA) database. The filtration of raw data (clean data) was mapped to the genome sequence of *C. roseus* through Hierarchical Indexing for Spliced Alignment of Transcripts (HiSAT2). The expression levels of all mapped reads were normalized by the transcripts per kilobase million (TPM) method.

Real-time quantitative PCR (RT-qPCR) used the housekeeping gene CrActin1 (CRO_01G008130.3) as an internal control. After 40 reaction cycles, the relative expression levels of CrLBD genes and MIA biosynthetic genes were verified. Primers used for RT-qPCR analysis were designed using Primer 5 software (Table S1). All RT-qPCR analyses were performed with three biological replicates and used the 2^−ΔΔCt^ method. After that, the gene expression levels were visualized with the homogenized method involving log_2_ (TPM + 1) using the heatmap in R v 3.6.3. Pearson’s correlation coefficient was analyzed using the cor() function in R v 3.6.3, and significant difference was tested at significance levels of 0.05, 0.01, and 0.001.

### 2.6. Plant Materials, Growth Conditions, and MIA Accumulation

The seeds of *C. roseus* were grown in a greenhouse, under temperature controlled at 25 °C and photosynthetically active radiation (PAR) of 1500 ± 50 μmol m^−2^ s^−1^, at the Key Laboratory of Plant Ecology, Northeast Forestry University, Harbin, China. The fresh leaves were harvested after four hours of treatment. All samples were frozen by liquid nitrogen immediately and stored at −80 °C. Each sample includes three biological duplicates.

The samples were extracted with 100% methanol; the extracts were then evaporated in a speed vac and re-dissolved in 500 µL of methanol. Samples of 10 µL were injected into the HPLC system (Alliance 2690, Waters, Milford, CT, USA) equipped with a Hypersil ODS C18 column (5 μm, 4.6 mm × 250 mm) to separate MIAs. The mobile phase was a 30:70 mixture of 5 mM pH 7.0 phosphate buffer and 100% methanol. The flow rate was 1 mL min^−1^, and the eluted peaks were detected by a photodiode array detector (PDA) with wavelengths of 280 nm (catharanthine) and 310 nm (vindoline).

### 2.7. Y1H Assays and Subcellular Localization

The coding sequences of *CrLBD4* and *CrLBD11* were PCR amplified by using cDNA as template and specific primers, CrLBD4-JG45-F and CrLBD4-JG45-R, to obtain the gene sequence with overlap region and then mobilized to binary vector pJG4-5 pre-digested with *Eco*R I/*Xho* I. The constructs pJG4-5-*CrLBD4* and pJG4-5-*CrLBD11* were generated. The 2000 bp upstream promoter regions of MIA biosynthetic genes in *C. roseus* were analyzed with PlantPAN4.0 (http://plantpan.itps.ncku.edu.tw, accessed on 26 February 2024) to investigate putative LOB *cis*-elements. Based on the analysis, the triple repeated motifs of “aattatTCCGGccgc” were PCR amplified and cloned into the pLacZi vector, resulting in the plasmids pLacZi-*cis*-element. Primers were designed according to promoter sequences listed in Table S1. The recombinant pLacZi-*cis*-element was co-transformed into the yeast strain EGY48 along with pJG4-5-*CrLBD4* and pJG4-5-*CrLBD11*. Positive colonies were selected on SD/-Ura/-Trp medium and then plated onto SD/-Ura/-Trp medium supplemented with X-gal and incubated at 30 °C for 24–48 h. Binding activity was assessed by observing blue color development in yeast cells [41].

The coding sequences (CDSs) of *CrLBD4* and *CrLBD11* were amplified using the special primers listed in Table S1. The CDSs of *CrLBD4* and *CrLBD11* were inserted into pBIGD-*eGFP* vector to produce pBIGD-*eGFP*-*CrLBD4* (pro35S::*eGFP*-*CrLBD4*) and pBIGD-*eGFP*-*CrLBD11* (pro35S::*eGFP*-*CrLBD11*) according to our previous report [42]. The constructed vectors and empty vectors were transformed into *Agrobacterium tumefaciens* (GV3101) to treat *Nicotiana benthamiana* (*N. benthamiana*) leaves, which were described previously [43]. After 48 h, we harvested the transfected leaves, and 1 mg/mL of DAPI (catalog 0671, Roche, Basel, Switzerland) solution was injected into the transfected leaves to observe the nucleus. The green fluorescent protein (GFP) signal was detected by laser confocal fluorescence microscopy (LSM 880, ZEISS, Oberkochen, Germany).

### 2.8. Statistical Analysis

Statistical analyses were carried out using the SPSS 29.0 (IBM Corp., Armonk, NY, USA) statistics program. Data were compared using Student’s *t*-test. *p* < 0.05 indicated by *, *p* < 0.01 indicated by **, *p* < 0.001 indicated by ***.

## 3. Results

### 3.1. Identification of the LBD Gene Family in C. roseus

Using the method described above, a total of 34 CrLBDs were identified from the *C. roseus* genome. All CrLBDs contain an HMM profile of the LOB domain. The search results were used to rename them as CrLBD1–CrLBD34 according to chromosomal location (Table S2). The CRO_01G006870.2 (CrLBD3) was the longest protein, containing 415 amino acid residues, and the molecular weight was 46.71 kDa. The shortest protein was CRO_05G011230.1 (CrLBD21), which has just 153 amino acid residues, and its molecular weight was 17.32 kDa. The predicted theoretical isoelectric points (pI) ranged from 4.7 (CRO_05G028200.1, CrLBD22) to 8.96 (CRO_05G030340.1, CrLBD25), and the number of exons ranged from one to three. All CrLBD proteins were predicted to be localized in the nucleus (Table S2).

### 3.2. Phylogenetic and Conservative Analysis of the CrLBD Gene Family

**Figure 1:**
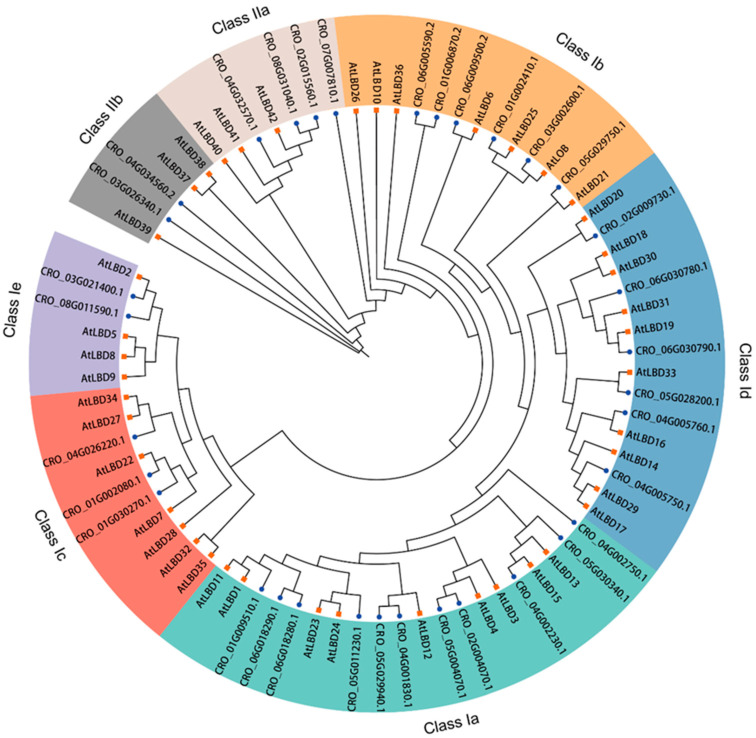
Unrooted phylogenetic tree representing relationships among the LBD proteins of *C. roseus* and *A. thaliana*. The tree divided the CrLBD proteins into seven subgroups represented by different colored clusters within the tree. The phylogenetic tree was derived using the ML method in IQ-TREE with 1000 bootstraps. The terminal branches of the phylogenetic tree connected to CrLBDs were marked with blue dots, while those connected to AtLBDs were marked with orange squares.

**Figure 2:**
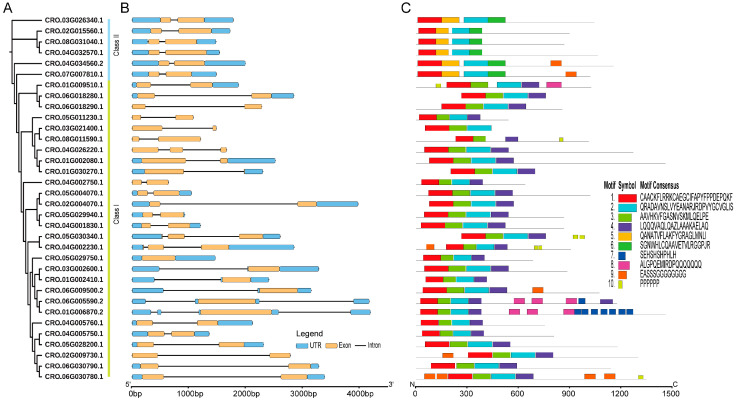
The phylogenetic relationships, conserved motifs, and gene structures of CrLBD gene family members. (**A**) An ML phylogenetic tree of *C. roseus* proteins was constructed in IQ-TREE with 1000 bootstraps. (**B**) The gene structures of the *CrLBD* genes include introns (black lines), exons (yellow rectangles), and UTR regions (blue rectangles). The scale bar indicates 1 kb. (**C**) Distribution of conserved motifs in the CrLBD proteins. The colored boxes represent motifs 1–10. The scale bar indicates 300 aa.

To understand the possible evolutionary relationship and to classify the CrLBD gene family, a maximum likelihood (ML) tree was constructed based on the full-length amino acid sequences of LBD proteins from *C. roseus* and *A. thaliana* (Figure 1). According to the phylogenetic tree results, a total of 77 LBD proteins from two species were phylogenetically categorized into seven subgroups (classes Ia, Ib, Ic, Id, and Ie and classes IIa and IIb). In *A. thaliana*, 9, 7, 7, 10, 4, 3, and 3 CrLBDs were clustered into the seven subgroups, respectively. In *C. roseus*, 11, 6, 3, 6, 2, 4, and 2 CrLBDs were clustered into seven subgroups, respectively. Based on the above statistics, it can be observed that among the members of Class I in the *C. roseus*, except for those in Class Ia, which showed an expansion trend, other members exhibited significant contraction. In Class II, the number of LBD members remained constant, possibly related to the more complex specialized metabolite biosynthesis in *C. roseus*. Notably, most sister pair clades consisted of CrLBDs and *A. thaliana* LBDs (AtLBDs) (e.g., CRO_05G029750.1/AtLBD21, CRO_03G002600.1/AtLOB, CRO_01G002410.1/AtLBD25, CRO_02G009730.1/AtLBD20, etc.). Notably, seven CrLBD proteins lack the LX6LX3LX6L leucine zipper-like coiled-coil motif (Figure S1). By constructing the phylogenetic tree of CrLBDs, it was found that six members were classified into the Class II subgroup, while member CRO_03G021400 was classified into the Class Ia subgroup (Figure 2A).

### 3.3. Gene Structure and Motif Composition of the CrLBD Gene Family

To determine differences in gene structure, a comparison of the exon and intron structures was conducted for 34 *CrLBD* genes; this analysis provides valuable evidence for the evolutionary diversity of the CrLBD family structure. Nearly all *CrLBD* genes contained two exons (Figure 2B and Table S2). In addition, the genes clustered into subgroups have similar gene structures, such as the extremely similar gene structures of all members of Class II (Figure 2B).

The MEME software was used to predict the conserved motifs of the 34 CrLBD proteins. A total of 10 conserved motifs were identified (Table S3). As the results show, the numbers and types of conserved motifs in CrLBD protein sequences were relatively conserved. Most CrLBD members clustered into the same subgroup had similar motif structures, suggesting that CrLBD proteins from the same subgroup may have similar functions. Motif 1, motif 2, motif 3, and motif 4 were basic LOB domain regions detected in nearly all members of the CrLBD gene family. Class I members had motif 1 (CX2CX6CX3C), motif 2 and motif 3 (GAS blocks), and motif 4 (LX6LX3LX6L), while motif 4 (LX6LX3LX6L) is missing from the Class II members and has been replaced by motif 5 (Figure 2C). This result provided further evidence to support dividing the CrLBD members into two clusters.

### 3.4. Chromosomal Distribution and Synteny Analysis of the CrLBD Gene Family

The 34 *CrLBD* genes were unevenly distributed on eight chromosomes, and the density of genes on each chromosome was also uneven (Figure S2). Among them, chromosome Chr4 had the most indispensable genes distributed. It contained eight *CrLBD* genes, followed by chromosomes Chr5 and Chr6, containing six *CrLBD* genes. The three gene pairs are closely linked with the chromosome (*CrLBD15*/*CrLBD16*, *CrLBD28*/*CrLBD29*, *CrLBD30*/*CrLBD31*) and belong to the *C. roseus* paralogous gene pair.

**Figure 3:**
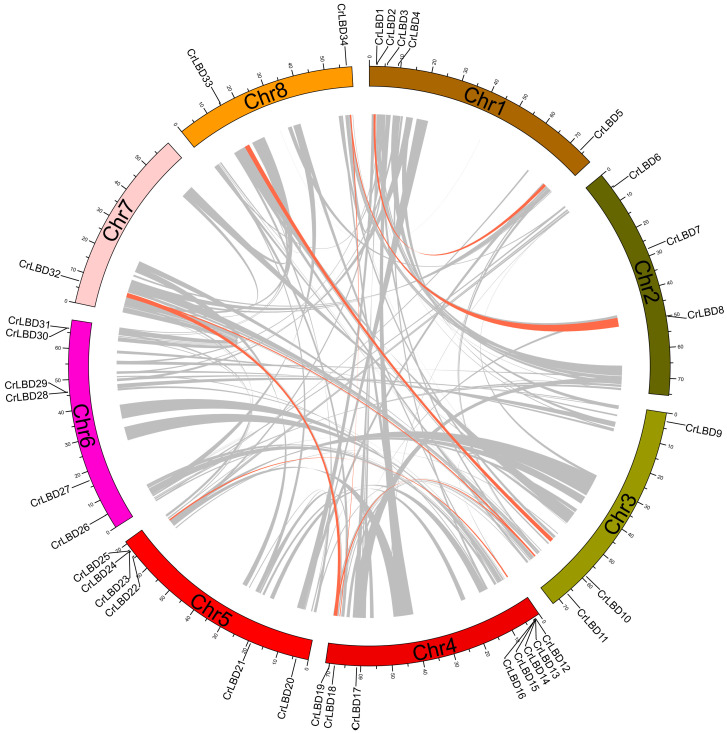
Synteny analysis of *CrLBD* genes in the *C. roseus* genome. Duplicated blocks in *C. roseus* chromosomes were revealed. The circular image shows inter-chromosome homologous regions connected by bands in different colors. The chromosome numbers and *CrLBD* genes are indicated outside. Gray lines represent all syntenic regions in the whole maize genome, and orange lines represent *CrLBD* gene pairs with segmental duplication.

Segmental and tandem duplications have been reported to be the main mechanisms of diversification of LBD gene families [44]. Duplication blocks in the *C. roseus* genome were detected using Circos v 0.69-8 software to analyze the duplication events of *CrLBD* genes. There are nine pairs of *CrLBD* genes, such as *CrLBD1*/*CrLBD5*, *CrLBD8*/*CrLBD34*, *CrLBD11*/*CrLBD19*, *CrLBD11*/*CrLBD32*, *CrLBD10*/*CrLBD33*, *CrLBD12*/*CrLBD24*, *CrLBD13*/*CrLBD25*, *CrLBD19*/*CrLBD32*, and *CrLBD18*/*CrLBD34*, that were located in duplicated genomic regions (Figure 3). We found that most *CrLBD* genes existed in gene clusters, suggesting that the CrLBD gene family members had undergone segmental or tandem duplication.

**Figure 4:**
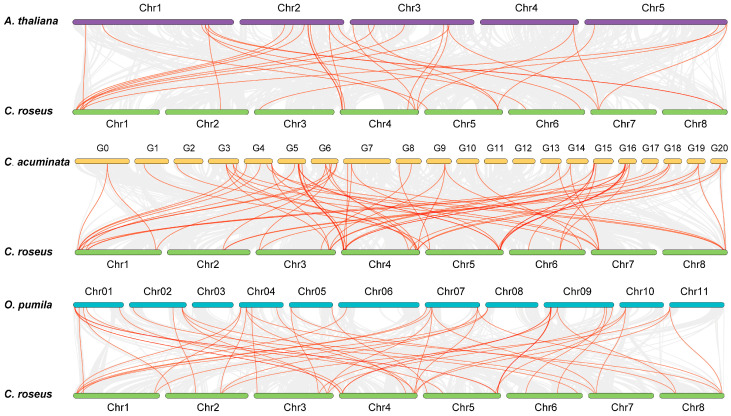
Synteny analysis of *CrLBD* genes between *C. roseus* and three dicotyledonous plants. Gray lines in the background indicate the collinear blocks within the *C. roseus* and other plant genomes; the orange lines highlight the syntenic *LBD* gene pairs.

To gain insights into the evolution of the CrLBDs, synteny analysis was conducted between *C. roseus* and three dicotyledonous plants (*A. thaliana*, *O. pumila*, and *C. acuminata*) (Figure 4). The red lines represent collinearity blocks among the LBD gene family members, and the gray lines represent collinearity blocks within and between genomes. In total, *CrLBD* genes displayed different syntenic relationships with *A. thaliana*, *O. pumila*, *C. acuminata*, and *C. roseus*, respectively. The results showed that the *LBD* genes were more frequently colinear relationships between *C. roseus*, *C. acuminata*, and *O. pumila*. This may be because the three plants can produce MIAs, making them more evolutionarily related. The collinearity information of the LBD family members is listed in Table S4.

### 3.5. Cis-Element Analysis in the Putative Promoter of CrLBD Genes

The *cis*-elements on the promoter regions can respond to inducible transcription factors, as revealed by the analysis of *cis*-elements in the promoter regions of 34 CrLBD gene family members (Figure S3). It was found that various endogenous signals related to plant hormone responses, growth regulation, and stress were present as *cis*-elements on the promoters. In response to plant hormones and growth regulation, seven *cis*-elements were detected on the promoters of the 34 CrLBD gene family members, whereas five types were detected in stress response. These findings suggest that *CrLBD* genes may be crucial in phytohormone signal transduction and stress response in *C. roseus.*

### 3.6. Potential Functions of CrLBDs in MIA Biosynthesis

**Figure 5:**
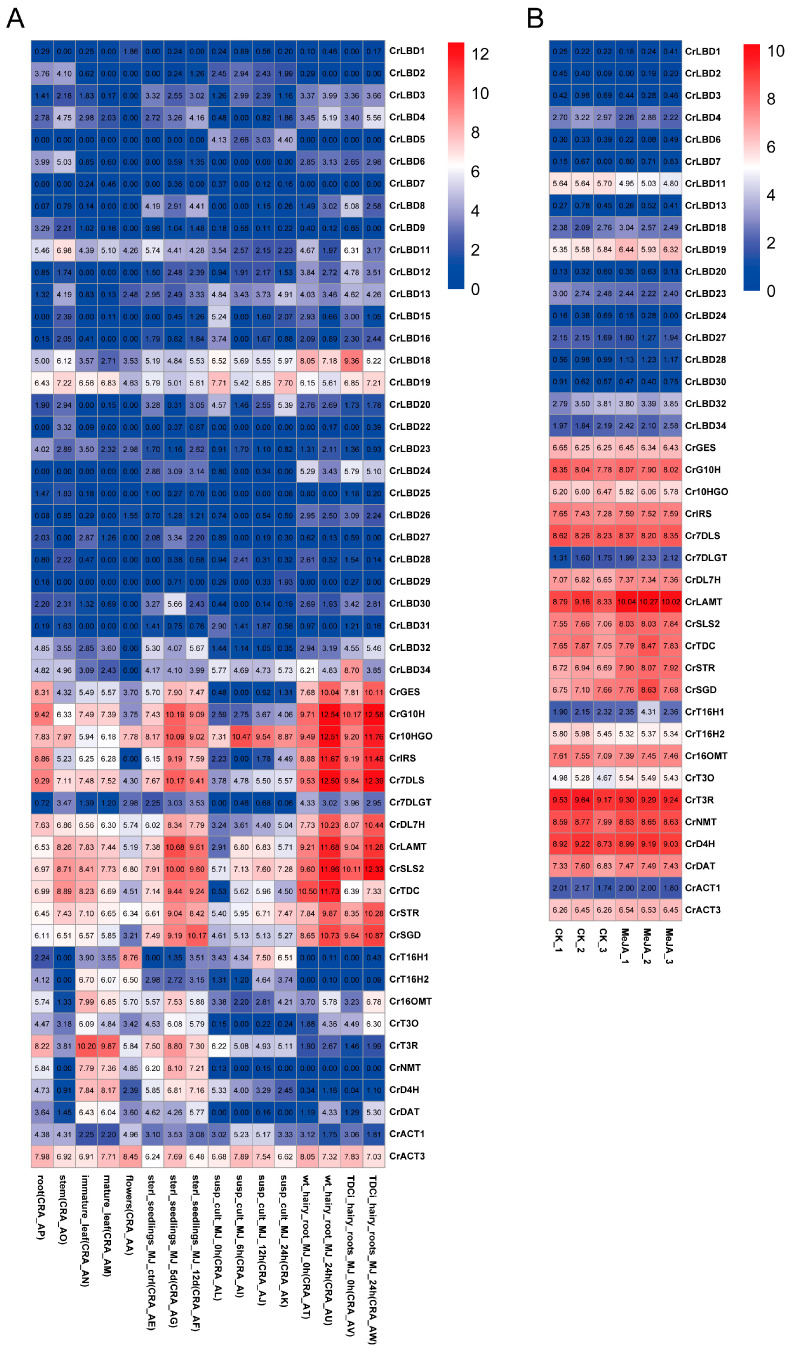
The expression patterns of *CrLBD* and MIA biosynthetic genes in different and MeJA-treated tissues. (**A**) Expression patterns of *CrLBD* and MIA biosynthetic genes in the transcriptomic data of SRP005953. (**B**) Expression patterns of *CrLBD* and MIA biosynthetic genes in the transcriptomic data of PRJNA358259. The color scale shows increasing expression levels from blue to red, and the expression patterns of *CrLBD* genes are shown on a heatmap using transcripts per kilobase million (TPM).

We studied the expression levels of CrLBD gene family members and MIA biosynthetic genes in *C. roseus* using published transcriptome data. Based on the latest genome information of *C. roseus*, TPM values were recalculated according to new gene IDs, and expression profiles were plotted with heatmaps (Figure 5). Transcriptome analysis of different tissues and methyl jasmonate (MeJA) treatments in *C. roseus* revealed that most MIA biosynthetic genes (*CrGES*, *CrG10H*, *Cr10HGO*, *CrIRS*, *Cr7DLS*, *CrDL7H*, *CrLAMT*, *CrSLS2*, *CrTDC*, *CrSTR*, and *CrSGD*) were significantly upregulated in the MeJA-treated group (Tables S5 and S6). Compared to the expression levels of MIA biosynthetic genes, the expression levels of most CrLBD gene family members were relatively low. *CrLBD10*, *CrLBD14*, *CrLBD17*, *CrLBD21*, and *CrLBD33* were not detected in any samples from the two transcriptomes.

**Figure 6:**
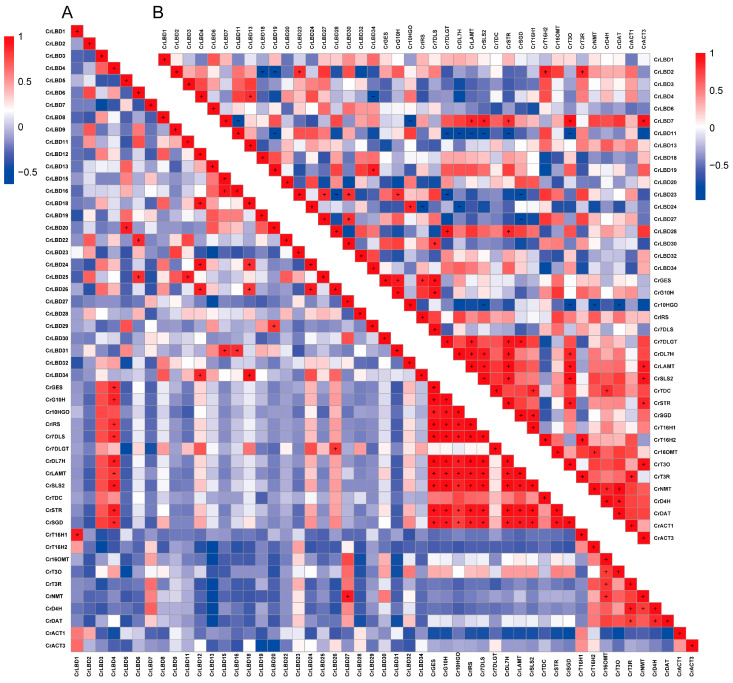
The correlation between the expression patterns of *CrLBD* and MIA biosynthetic genes. (**A**) Pearson correlation of *CrLBD* and MIA biosynthetic genes in the transcriptomic data of SRP005953. (**B**) Pearson correlation of *CrLBD* and MIA biosynthetic genes in the transcriptomic data of PRJNA358259. Red: positively correlated, the “+” indicates that the Pearson correlation coefficient is greater than 0.8; blue: negatively correlated, the “−” indicates that the Pearson correlation coefficient is less than −0.8.

In addition, the correlation between CrLBD gene family members and MIA biosynthetic genes was analyzed (Figure 6), with Pearson correlation coefficients greater than 0.8 indicated by “+” and less than −0.8 indicated by “−.” The results showed significant correlations in the expression levels of MIA biosynthetic genes in the iridoid metabolic pathway; MIA biosynthetic genes in the vindoline biosynthesis pathway also exhibited significant correlations, consistent with previous reports [45]. Further observations revealed that CrLBD4 showed a significant positive correlation with MIA biosynthetic genes in the iridoid metabolic pathway (*p* < 0.05, r ≥ 0.8), and CrLBD3 exhibited a similar positive correlation (*p* < 0.05, r > 0.7). CrLBD11, on the other hand, showed a significant negative correlation with MIA biosynthetic genes in the iridoid metabolic pathway (*p* < 0.05, r ≤ −0.8). Previous studies have found that AtLOB (AtLBD) can regulate proximal-distal patterning in Arabidopsis petals, while AtLBD11 can promote secondary growth in plants [46,47]. In *Catharanthus roseus*, CrLBD4, as a homolog of AtLOB and AtLBD11, may also have potential regulatory functions in the growth and development of *C. roseus*, as well as potential roles in regulating the plant’s specialized metabolism [48,49]. *C. roseus* CrLBD11 belongs to the Class IIb subfamily, and its homologs are AtLBD37, AtLBD38, and AtLBD39. The Class IIb subgroup members in various plants are involved in anthocyanin biosynthesis and nitrate metabolism [22,50]. This also suggests that CrLBD11 is likely involved in the specialized metabolism of *C. roseus*. Jasmonic acid can significantly induce the expression of MIA biosynthetic genes in *C. roseus* and promote the accumulation of their products [30]. Additionally, it has been found that transcription factors such as ORCA3 can respond to MeJA signals to regulate the synthesis of vindoline and catharanthine. Through transcriptome and co-expression analysis, genes such as *CrLBD4* and *CrLBD11*, which respond to MeJA signaling, were identified as potential participants in the MIA biosynthesis process in *C. roseus*.

**Figure 7:**
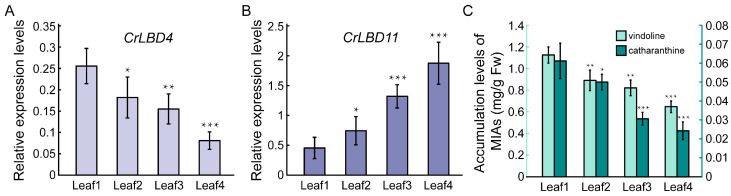
Relative expression levels of *CrLBD4/11* and MIA accumulation levels in different developmental stages of *C. roseus* leaves. (**A**) Relative expression of *CrLBD4* in different developmental stages of *C. roseus* leaves by RT-qPCR analysis, *p* < 0.05, *p* < 0.01. (**B**) Relative expression of *CrLBD11* in different developmental stages of *C. roseus* leaves by RT-qPCR analysis, *p* < 0.05, *p* < 0.01. Results were normalized to *CrActin1*, the error bars represent standard errors from three biological replicates, and each replicate contains five leaves from five independent plants with the same size and developmental age. (**C**) MIA accumulation levels in different developmental stages of leaves with 4-week-old *C. roseus* plants (*p* < 0.05 indicated by *, *p* < 0.01 indicated by **, *p* < 0.01 indicated by ***).

Based on previous reports, the expression levels of iridoid biosynthetic genes and the accumulation levels of vindoline and catharanthine in different developmental stages of *C. roseus* leaves show a gradually decreasing trend [51]. According to the results in Figure 6, the expression patterns of *CrLBD4* are positively correlated with the iridoid biosynthetic genes, while *CrLBD11* is negatively correlated. Subsequently, we used RT-qPCR to detect the expression patterns of *CrLBD4* and *CrLBD11* in different developmental stages of *C. roseus* leaves. The results showed that the expression levels of *CrLBD4* were high in Leaf1 (immature leaf) and significantly decreased as the leaves matured (Leaf4, mature leaf) (Figure 7A). This trend is consistent with the accumulation levels of vindoline and catharanthine in leaves (Figure 7C). In contrast, the expression levels of *CrLBD11* gradually increased as the leaves matured (Figure 7B). These results further demonstrate that *CrLBD4* and *CrLBD11* are correlated with the expression levels of iridoid biosynthetic genes and the accumulation levels of vindoline and catharanthine. Thus, *CrLBD4* and *CrLBD11* may be involved in the MIA biosynthesis process in *C. roseus*.

### 3.7. CrLBD4/11 as Transcription Factors Might Regulate MIA Biosynthetic Genes

**Figure 8:**
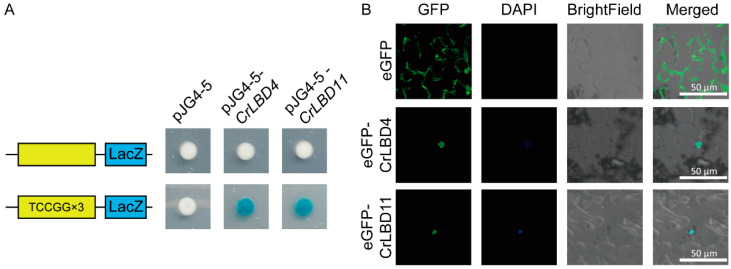
CrLBD4/11 as transcription factors might regulate MIA biosynthetic genes. (**A**) Y1H assays show that CrLBD4/11 binds to triple-repeated motifs of “aattatTCCGGccgc.” (**B**) Subcellular localization of CrLBD4/11 in *N. benthamiana* leaf epidermal cells. Co-localization of GFP and DAPI was assessed in overlay pictures (Merged). Scale bars: 50 μm.

Using PlantPAN4.0, we analyzed the 2000 bp promoter sequences of MIA biosynthetic genes (Figure S4). The prediction results indicated that CrLBD gene family members have potential binding sites in the promoter regions of MIA biosynthetic genes such as *Cr7DLGT*, *Cr10HGO*, *CrNMT*, *CrSLS2*, *CrSTR*, *CrT16H1*, and *CrTDC* (Table S7). We selected the *cis*-elements (atTCCGG, from *Cr7DLGT*) located closest to the promoter start site and with the highest similarity scores. To investigate whether CrLBD4 and CrLBD11 could bind to the identified *cis*-element, triple repeated motifs of “aattatTCCGGccgc” were selected for Y1H assays. The results demonstrated that the CrLBD4 and CrLBD11 could bind to the “aattatTCCGGccgc” *cis*-element in the *Cr7DLGT* promoter regions, suggesting direct regulation of CrLBD4 and CrLBD11 on the expression of *Cr7DLGT* and in *C. roseus* (Figure 8A).

Furthermore, CrLBD4 and CrLBD11 were selected for further subcellular localization. To observe the subcellular localization of CrLBD4 and CrLBD11, the GFP gene was fused to the C-terminus of the CrLBD4/11 protein driven by the 35S promoter and expressed in tobacco leaves. In Figure 8B, we observed that 35S::*eGFP* was located at the membrane and nucleus, while 35S::*CrLBD4-eGFP* and 35S::*CrLBD11-eGFP* showed strong fluorescence signals in the nucleus. This result confirms that CrLBD4 and CrLBD11 are transcription factors (Figure 8B). Summarizing the above results, we hypothesize that CrLBD4 and CrLBD11, as transcription factors, may be involved in regulating MIA genes.

## 4. Discussion

Transcription factors are crucial regulatory elements in nearly all plant life processes, including the biosynthesis of MIAs in *C. roseus*. For instance, members of the AP2/ERF superfamily in *C. roseus*, specifically ORCA2 and ORCA3, positively regulate the expression of genes in the indole and iridoid pathways in response to jasmonic acid signals [30,52,53]. Additionally, MYC2, a transcription factor from the bHLH family, is regulated by jasmonic acid signals and directly binds to the JRE motif of the ORCA3 promoter, thereby activating ORCA3 expression [32]. Another transcription factor, WRKY1, enhances MIA biosynthesis when regulated by jasmonic acid, gibberellin, and ethylene [31]. While new members of these gene families are continually being discovered, which is crucial for regulating MIA biosynthesis, current research predominantly focuses on a few key members, leaving the study of new family members in its infancy [54,55].

Transcription factor families are numerous in plants, and many gene families in *C. roseus* have yet to be thoroughly analyzed and described. The LBD gene family is a plant-specific transcription factor family. Functional validation of these genes has shown that Class I primarily participates in growth and developmental regulation. At the same time, Class II may be involved in responses to environmental stress and specialized metabolism [56,57,58]. Phylogenetic analysis reveals that LBD proteins in *C. roseus* and *A. thaliana* are relatively conserved throughout evolution, suggesting that proteins clustered in the same subgroup may have similar functions (Figure 1). As previously reported, members of the Class IIb subgroup in *A. thaliana* (AtLBD37, AtLBD38, and AtLBD39) act as negative regulators in anthocyanin biosynthesis [22,26]. Experimental results indicate that CrLBD11 is a member of the Class IIb subgroup, and its gene expression level is inversely correlated with the accumulation of metabolic products. Additionally, co-expression analysis suggests that CrLBD4, a member of the Class I subgroup, may have a potential role in regulating MIA biosynthesis in *C. roseus*. When the selection Pearson correlation coefficient is relaxed to “±0.6”, Class I subgroup members CrLBD3, CrLBD24, and CrLBD26 also emerge as candidate genes for further study. Furthermore, we found that CrLBD27, another Class I subgroup member, exhibits high co-expression with biosynthetic genes involved in the vindoline metabolic pathway. These findings, combined with previous work, suggest that genes within Class I may have significant implications for specialized metabolic functions.

In *C. roseus*, MIA biosynthetic genes demonstrate complex patterns of cell-type-specific expression in various cells, such as inner phloem-associated parenchyma (IPAP), epidermis, and a specialized cell type called an idioblast [59,60,61]. These genes are not only present in different cell types but are also spread across various organelles. Tetrahydroalstonine synthase (THAS) is an MIA biosynthetic gene found in the nucleus of *C. roseus*, suggesting that its nuclear localization might be related to interactions with upstream enzymes or the regulation of gene expression linked to alkaloid biosynthesis [62]. Both CrLBD4 and CrLBD11, identified in the nucleus through subcellular localization studies, present an opportunity to explore the regulatory relationships between CrLBD4, CrLBD11, and THAS, as well as to analyze their expression patterns with MIA biosynthetic genes in specific cells using single-cell sequencing.

Completing plant genomic information is crucial for gene discovery and the exploration of their biological functions. When analyzing the LBD gene family using version 2 of the *C. roseus* genome, 33 members were identified. However, using version 3 of the genome, 34 LBD gene family members were found, one more than in version 2 (Figure S5). The karyotype maps from the two versions reveal that incomplete genomic information can lead to the underestimation of gene numbers and the loss of chromosomal information. For example, only two gene pairs were identified in version 2. This difference highlights the importance of using comprehensive and updated genomic data for accurate gene identification and mapping.

Overall, the potential functions of *LBD* genes require further experiments to explore and confirm. Our research provides new insights for further studies on the role of LBD gene family members in regulating MIA biosynthesis in *C. roseus*. It offers a theoretical foundation for their utilization in production.

## 5. Conclusions

In this study, the LBD gene family in *C. roseus* was systematically identified and characterized. A comparison of the gene structure, chromosome distribution, phylogenetic relationships, compositional features, and *cis*-elements of 34 CrLBDs was conducted. The expression patterns and co-expression analysis highlight the potential roles of CrLBD4/11 in regulating the MIA biosynthesis of *C. roseus*. Y1H and subcellular localization assays demonstrated that CrLBD4 and CrLBD11 as transcription factors might regulate MIA biosynthetic genes. This research added important information about *LBD* genes and provided new insights to explore the molecular mechanisms by which the CrLBD4/11 might regulate MIA biosynthesis in *C. roseus.*

## Data Availability

The genomic information of *C. roseus* was downloaded from the DRYAD database (https://doi.org/10.5061/dryad.d2547d851, accessed on 18 February 2024). The raw data sequencing data used during this study were obtained from NCBI (https://www.ncbi.nlm.nih.gov/sra/, accessed on 28 February 2024) under accession numbers PRJNA358259 and SRP005953, respectively. All data generated or analyzed during this study are included in this published article and its Supplementary Information files.

## Supplementary Materials

The following supporting information can be downloaded at https://www.mdpi.com/article/10.3390/genes15091140/s1. Figure S1: Multiple sequence alignment of LBD gene family in *C. roseus*; Figure S2: Chromosome distribution of 34 LBD gene family members in *C. roseus* (version 3); Figure S3: The *cis*-elements on putative promoters of CrLBD gene family members; Figure S4: The *cis*-elements in the upstream of MIA pathway genes in *C. roseus*; Figure S5: Chromosome distribution of LBD gene family members in *C. roseus* (version 2); Table S1: Primers used in this study; Table S2: Characteristic features of LBD gene family identified in *C. roseus*; Table S3: Conserved motifs of the LBD proteins in *C. roseus*; Table S4: One-to-one orthologous relationships between *C. roseus* and *A. thaliana*, *C. roseus* and *C. acuminata*, and *C. roseus and O. pumila*; Table S5: Expression matrix of *CrLBD* and MIA biosynthetic genes in SRP005953; Table S6: Expression matrix of *CrLBD* and MIA biosynthetic genes in PRJNA358259; Table S7: Prediction of CrLBD transcription factor binding sites in different MIA gene promoters using PlantPAN4.0.

## Abbreviations

*A. thaliana*: *Arabidopsis thaliana*; AtLBD: *A. thaliana* LBD; CDD: Conserved Domain Database; CDS: coding sequence; CrLBD: *C. roseus* LBD; *C*. *roseus*: *Catharanthus roseus*; DIECA: sodium diethyldithiocarbamate trihydrate; eGFP: enhanced green fluorescent protein; GAS: Gly-Ala-Ser; HiSAT2: Hierarchical Indexing for Spliced Alignment of Transcripts; HMM: Hidden Markov Model; IPAP: inner phloem-associated parenchyma; LBD: Lateral Organ Boundaries Domain; LOBs: Lateral Organ Boundaries; MeJA: methyl jasmonate; MIAs: monoterpenoid indole alkaloids; ML: maximum likelihood; MPGR: Medicinal Plant Genomics Resource databases; MW: molecular weight; *N. benthamiana*: *Nicotiana benthamiana*; NCBI: National Center for Biotechnology Information; PAR: photosynthetically active radiation; PDA: photodiode array detector; pI: theoretical isoelectric points; RT-qPCR: real-time quantitative PCR; SMART tool: Simple Modular Architecture Research Tool; SRA: Sequence Read Archive; TFs: transcription factors; THAS: tetrahydroalstonine synthase; TPM: transcripts per kilobase million.

## References

[1] Kim J., Buell C.R. (2015). A Revolution in Plant Metabolism: Genome-Enabled Pathway Discovery. Plant Physiol..

[2] Nutzmann H.W., Huang A., Osbourn A. (2016). Plant metabolic clusters—From genetics to genomics. New Phytol..

[3] Zhan C., Shen S., Yang C., Liu Z., Fernie A.R., Graham I.A., Luo J. (2022). Plant metabolic gene clusters in the multi-omics era. Trends Plant Sci..

[4] Li C., Wood J.C., Vu A.H., Hamilton J.P., Rodriguez Lopez C.E., Payne R.M.E., Serna Guerrero D.A., Gase K., Yamamoto K., Vaillancourt B. (2023). Single-cell multi-omics in the medicinal plant *Catharanthus roseus*. Nat. Chem. Biol..

[5] Gladman N., Goodwin S., Chougule K., Richard McCombie W., Ware D. (2023). Era of gapless plant genomes: Innovations in sequencing and mapping technologies revolutionize genomics and breeding. Curr. Opin. Biotechnol..

[6] Satam H., Joshi K., Mangrolia U., Waghoo S., Zaidi G., Rawool S., Thakare R.P., Banday S., Mishra A.K., Das G. (2023). Next-Generation Sequencing Technology: Current Trends and Advancements. Biology.

[7] Wafula E.K., Zhang H., Von Kuster G., Leebens-Mack J.H., Honaas L.A., de Pamphilis C.W. (2022). PlantTribes2: Tools for comparative gene family analysis in plant genomics. Front. Plant Sci..

[8] Chen C., Chen X., Han J., Lu W., Ren Z. (2020). Genome-wide analysis of the WRKY gene family in the cucumber genome and transcriptome-wide identification of WRKY transcription factors that respond to biotic and abiotic stresses. BMC Plant Biol..

[9] Ayaz A., Saqib S., Huang H., Zaman W., Lü S., Zhao H. (2021). Genome-wide comparative analysis of long-chain acyl-CoA synthetases (LACSs) gene family: A focus on identification, evolution and expression profiling related to lipid synthesis. Plant Physiol. Biochem..

[10] Chakraborty P., Biswas A., Dey S., Bhattacharjee T., Chakrabarty S. (2023). Cytochrome P450 Gene Families: Role in Plant Secondary Metabolites Production and Plant Defense. J. Xenobiotics.

[11] Feng J.-X., Liu D., Pan Y., Gong W., Ma L.-G., Luo J.-C., Deng X.W., Zhu Y.-X. (2005). An annotation update via cDNA sequence analysis and comprehensive profiling of developmental, hormonal or environmental responsiveness of the Arabidopsis AP2/EREBP transcription factor gene family. Plant Mol. Biol..

[12] Huang J., Gu M., Lai Z., Fan B., Shi K., Zhou Y.H., Yu J.Q., Chen Z. (2010). Functional analysis of the Arabidopsis PAL gene family in plant growth, development, and response to environmental stress. Plant Physiol..

[13] Liu S., Meng Z., Zhang H., Chu Y., Qiu Y., Jin B., Wang L. (2022). Identification and characterization of thirteen gene families involved in flavonoid biosynthesis in Ginkgo biloba. Ind. Crops Prod..

[14] Li C., Zhu S., Zhang H., Chen L., Cai M., Wang J., Chai J., Wu F., Cheng Z., Guo X. (2017). OsLBD37 and OsLBD38, two class II type LBD proteins, are involved in the regulation of heading date by controlling the expression of Ehd1 in rice. Biochem. Biophys. Res. Commun..

[15] Xu C., Luo F., Hochholdinger F. (2016). LOB domain proteins: Beyond lateral organ boundaries. Trends Plant Sci..

[16] Yordanov Y.S., Regan S., Busov V. (2010). Members of the LATERAL ORGAN BOUNDARIES DOMAIN transcription factor family are involved in the regulation of secondary growth in Populus. Plant Cell.

[17] Cho C., Jeon E., Pandey S.K., Ha S.H., Kim J. (2019). LBD13 positively regulates lateral root formation in Arabidopsis. Planta.

[18] Lee H.W., Kim N.Y., Lee D.J., Kim J. (2009). LBD18/ASL20 regulates lateral root formation in combination with LBD16/ASL18 downstream of ARF7 and ARF19 in Arabidopsis. Plant Physiol..

[19] Shuai B., Reynaga-Pena C.G., Springer P.S. (2002). The lateral organ boundaries gene defines a novel, plant-specific gene family. Plant Physiol..

[20] Lee H.W., Kim M.J., Kim N.Y., Lee S.H., Kim J. (2013). LBD18 acts as a transcriptional activator that directly binds to the EXPANSIN14 promoter in promoting lateral root emergence of Arabidopsis. Plant J..

[21] Xiong J., Feng X., Zhang W., Wang X., Hu Y., Zhang X., Wu F., Guo W., Xie W., Wang Q. (2021). Class II LBD genes ZmLBD5 and ZmLBD33 regulate gibberellin and abscisic acid biosynthesis. bioRxiv.

[22] Rubin G., Tohge T., Matsuda F., Saito K., Scheible W.R. (2009). Members of the LBD family of transcription factors repress anthocyanin synthesis and affect additional nitrogen responses in Arabidopsis. Plant Cell.

[23] Liu W., Yu J., Ge Y., Qin P., Xu L. (2018). Pivotal role of LBD1 6 in root and root-like organ initiation. Cell. Mol. Life Sci..

[24] Lu X.-Y., Liang X.-Y., Li X., Shen P.-X., Cao X.-Y., Chen C., Song S.-H., Wang D.-H., Wang Z.-Z., Zhang Y. (2020). Genome-wide characterisation and expression profiling of the LBD family in *Salvia miltiorrhiza* reveals the function of LBD50 in jasmonate signaling and phenolic biosynthesis. Ind. Crops Prod..

[25] Zeng J., Wang J., Liu X., Qin J., Lan X., Chen M., Liao Z. (2020). An auxin-responsive transcription factor AbLBD1 promotes the development of lateral roots and reduces the biosynthesis of tropane alkaloids in *Atropa belladonna*. Plant Cell Tissue Organ Cult. (PCTOC).

[26] Feng Z., Zhu J., Du X., Cui X. (2012). Effects of three auxin-inducible LBD members on lateral root formation in *Arabidopsis thaliana*. Planta.

[27] Ma Y., Wang F., Guo J., Zhang X.S. (2009). Rice OsAS2 gene, a member of LOB domain family, functions in the regulation of shoot differentiation and leaf development. J. Plant Biol..

[28] Wang X., Zhang S., Su L., Liu X., Hao Y. (2013). A genome-wide analysis of the LBD (LATERAL ORGAN BOUNDARIES domain) gene family in *Malus domestica* with a functional characterization of MdLBD11. PLoS ONE.

[29] Yu F., De Luca V. (2013). ATP-binding cassette transporter controls leaf surface secretion of anticancer drug components in *Catharanthus roseus*. Proc. Natl. Acad. Sci. USA.

[30] van der Fits L., Memelink J. (2000). ORCA3, a jasmonate-responsive transcriptional regulator of plant primary and secondary metabolism. Science.

[31] Suttipanta N., Pattanaik S., Kulshrestha M., Patra B., Singh S.K., Yuan L. (2011). The transcription factor CrWRKY1 positively regulates the terpenoid indole alkaloid biosynthesis in *Catharanthus roseus*. Plant Physiol..

[32] Zhang H., Hedhili S., Montiel G., Zhang Y., Chatel G., Pre M., Gantet P., Memelink J. (2011). The basic helix-loop-helix transcription factor CrMYC2 controls the jasmonate-responsive expression of the ORCA genes that regulate alkaloid biosynthesis in *Catharanthus roseus*. Plant J..

[33] Garcia-Hernandez M., Berardini T., Chen G., Crist D., Doyle A., Huala E., Knee E., Lambrecht M., Miller N., Mueller L.A. (2002). TAIR: A resource for integrated Arabidopsis data. Funct. Integr. Genom..

[34] Altschul S.F., Gish W., Miller W., Myers E.W., Lipman D.J. (1990). Basic local alignment search tool. J. Mol. Biol..

[35] El-Gebali S., Mistry J., Bateman A., Eddy S.R., Luciani A., Potter S.C., Qureshi M., Richardson L.J., Salazar G.A., Smart A. (2019). The Pfam protein families database in 2019. Nucleic Acids Res..

[36] Gasteiger E., Gattiker A., Hoogland C., Ivanyi I., Appel R.D., Bairoch A. (2003). ExPASy: The proteomics server for in-depth protein knowledge and analysis. Nucleic Acids Res..

[37] Voorrips R. (2002). MapChart: Software for the graphical presentation of linkage maps and QTLs. J. Hered..

[38] Chou K.-C., Shen H.-B. (2010). Cell-PLoc 2.0: An improved package of web-servers for predicting subcellular localization of proteins in various organisms. Nat. Sci..

[39] Hu B., Jin J., Guo A.-Y., Zhang H., Luo J., Gao G. (2015). GSDS 2.0: An upgraded gene feature visualization server. Bioinformatics.

[40] Bailey T.L., Johnson J., Grant C.E., Noble W.S. (2015). The MEME suite. Nucleic Acids Res..

[41] Xu M., Wu C., Zhao L., Wang Y., Wang C., Zhou W., Ming Y., Kai G. (2020). WRKY transcription factor OpWRKY1 acts as a negative regulator of camptothecin biosynthesis in *Ophiorrhiza pumila* hairy roots. Plant Cell Tissue Organ Cult. (PCTOC).

[42] Wang Y., Wang Y., Bai H., Han Y., Yu F. (2022). An ABCG-Type Transporter Facilitates ABA Influx and Regulates Camptothecin Biosynthesis in *Camptotheca acuminata*. Int. J. Mol. Sci..

[43] Chang C., Liu Z., Wang Y., Tang Z., Yu F. (2019). A bZIP transcription factor, CaLMF, mediated light-regulated camptothecin biosynthesis in *Camptotheca acuminata*. Tree Physiol..

[44] Jiang S.-Y., González J.M., Ramachandran S. (2013). Comparative genomic and transcriptomic analysis of tandemly and segmentally duplicated genes in rice. PLoS ONE.

[45] Larsen B., Fuller V.L., Pollier J., Van Moerkercke A., Schweizer F., Payne R., Colinas M., O’Connor S.E., Goossens A., Halkier B.A. (2017). Identification of iridoid glucoside transporters in *Catharanthus roseus*. Plant Cell Physiol..

[46] Chalfun-Junior A., Franken J., Mes J.J., Marsch-Martinez N., Pereira A., Angenent G.C. (2005). ASYMMETRIC LEAVES2-LIKE1 gene, a member of the AS2/LOB family, controls proximal–distal patterning in Arabidopsis petals. Plant Mol. Biol..

[47] Ha C.M., Jun J.H., Nam H.G., Fletcher J.C. (2007). BLADE-ON-PETIOLE1 and 2 control Arabidopsis lateral organ fate through regulation of LOB domain and adaxial-abaxial polarity genes. Plant Cell.

[48] Dang T.V.T., Lee S., Cho H., Choi K., Hwang I. (2023). The LBD11-ROS feedback regulatory loop modulates vascular cambium proliferation and secondary growth in Arabidopsis. Mol. Plant.

[49] Ye L., Wang X., Lyu M., Siligato R., Eswaran G., Vainio L., Blomster T., Zhang J., Mähönen A.P. (2021). Cytokinins initiate secondary growth in the Arabidopsis root through a set of LBD genes. Curr. Biol..

[50] Zhu X., Wang D., Xie L., Zhou T., Zhao J., Zhang Q., Yang M., Wu W., Lian X. (2022). Rice transcription factors OsLBD37/38/39 regulate nitrate uptake by repressing OsNRT2. 1/2.2/2.3 under high-nitrogen conditions. Crop J..

[51] Salim V., Yu F., Altarejos J., De Luca V. (2013). Virus-induced gene silencing identifies *Catharanthus roseus* 7-deoxyloganic acid-7-hydroxylase, a step in iridoid and monoterpene indole alkaloid biosynthesis. Plant J..

[52] Menke F.L., Champion A., Kijne J.W., Memelink J. (1999). A novel jasmonate- and elicitor-responsive element in the periwinkle secondary metabolite biosynthetic gene Str interacts with a jasmonate- and elicitor-inducible AP2-domain transcription factor, ORCA2. EMBO J..

[53] Van Der Fits L., Memelink J. (2001). The jasmonate-inducible AP2/ERF-domain transcription factor ORCA3 activates gene expression via interaction with a jasmonate-responsive promoter element. Plant J..

[54] Pan Q., Wang C., Xiong Z., Wang H., Fu X., Shen Q., Peng B., Ma Y., Sun X., Tang K. (2019). CrERF5, an AP2/ERF Transcription Factor, Positively Regulates the Biosynthesis of Bisindole Alkaloids and Their Precursors in *Catharanthus roseus*. Front. Plant Sci..

[55] Liu J., Gao F., Ren J., Lu X., Ren G., Wang R. (2017). A Novel AP2/ERF Transcription Factor CR1 Regulates the Accumulation of Vindoline and Serpentine in *Catharanthus roseus*. Front. Plant Sci..

[56] Huang B., Huang Z., Ma R., Ramakrishnan M., Chen J., Zhang Z., Yrjälä K. (2021). Genome-wide identification and expression analysis of LBD transcription factor genes in Moso bamboo (*Phyllostachys edulis*). BMC Plant Biol..

[57] Kim M., Kim M.-J., Pandey S., Kim J. (2016). Expression and protein interaction analyses reveal combinatorial interactions of LBD transcription factors during Arabidopsis pollen development. Plant Cell Physiol..

[58] Xiong J., Zhang W., Zheng D., Xiong H., Feng X., Zhang X., Wang Q., Wu F., Xu J., Lu Y. (2022). ZmLBD5 increases drought sensitivity by suppressing ROS accumulation in arabidopsis. Plants.

[59] Burlat V., Oudin A., Courtois M., Rideau M., St-Pierre B. (2004). Co-expression of three MEP pathway genes and geraniol 10-hydroxylase in internal phloem parenchyma of *Catharanthus roseus* implicates multicellular translocation of intermediates during the biosynthesis of monoterpene indole alkaloids and isoprenoid-derived primary metabolites. Plant J..

[60] Guirimand G., Guihur A., Poutrain P., Héricourt F., Mahroug S., St-Pierre B., Burlat V., Courdavault V. (2011). Spatial organization of the vindoline biosynthetic pathway in *Catharanthus roseus*. J. Plant Physiol..

[61] Miettinen K., Dong L., Navrot N., Schneider T., Burlat V., Pollier J., Woittiez L., Van Der Krol S., Lugan R., Ilc T. (2014). The seco-iridoid pathway from *Catharanthus roseus*. Nat. Commun..

[62] Stavrinides A., Tatsis E.C., Foureau E., Caputi L., Kellner F., Courdavault V., O’Connor S.E. (2015). Unlocking the diversity of alkaloids in *Catharanthus roseus*: Nuclear localization suggests metabolic channeling in secondary metabolism. Chem. Biol..

