# Supplementary material for: Genome-Wide Survey of the Potential Function of CrLBDs in Catharanthus roseus MIA Biosynthesis
Source: Genes (Basel). 2024 Aug 29;15(9):1140. doi: 10.3390/genes15091140 (PMC11431567; doi:10.3390/genes15091140)
Supplement: Supplementary file 1 [file genes-15-01140-s001.zip › Supplemental Figures.pdf]

Figure legends

Figure S1. Multiple sequence alignment of LBD gene family in *C. roseus*. Multiple sequence alignments show the CX2CX6CX3C zinc finger-like motif, GAS (Gly-Ala-Ser) block, and LX6LX3LX6L leucine zipper-like coiled-coil motif.

Figure S2. Chromosome distribution of 34 LBD gene family members in *C. roseus* (version 3). Chromosomal mapping was based on the physical position of 8 *C. roseus* chromosomes. The scale on the left indicates the size of the chromosomes (Mb). The chromosome numbers are displayed at the top of each bar. The three pairs of genes are marked in red.

Figure S3. The *cis*-elements on putative promoters of CrLBD gene family members. (A) A phylogenetic tree of the CrLBD protein was constructed using the ML method and 1000 bootstraps. The green-yellow branches of the phylogenetic tree indicate LBD genes in class I; the blue branches indicate LBD genes in class II. The Schematic of the CrLBD gene family promoter with *cis*-element involved in MeJA responsiveness and the positions of potential elements are shown as blue bars. (B) We have compiled statistics on 18 *cis*-elements from three aspects: biotic or abiotic stress, plant hormones, and plant growth, as well as the distribution and number of these elements among various promoters of LBD gene family members. The phylogenetic trees in Figures A and B show the names and IDs of the LBD gene family members in corresponding positions.

Figure S4. The *cis*-elements in the upstream of MIAs pathway genes in *C. roseus*. Schematic of the MIAs pathway genes promoter with *cis*-element involved in CrLBD responsiveness, the positions of potential elements are shown as colored bars. All potential *cis*-elements were predicted using the PlantPAN4.0 website.

Figure S5. Chromosome distribution of LBD gene family members in *C. roseus* (version 2). Chromosomal mapping was based on the physical position in 24 *C. roseus* scaffolds. The scale on the left is in megabases (Mb). The scaffold numbers are indicated at the top of each bar. The two pairs of genes are marked in red.

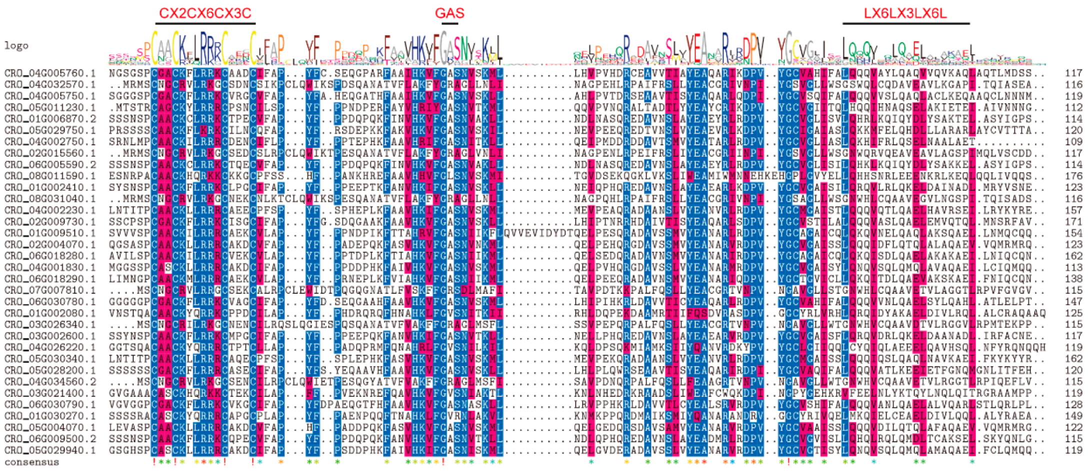

Figure S1. Multiple sequence alignment of LBD gene family in *C. roseus*. Multiple sequence alignments show the CX2CX6CX3C zinc finger-like motif, GAS (Gly-Ala-Ser) block, and LX6LX3LX6L leucine zipper-like coiled-coil motif.

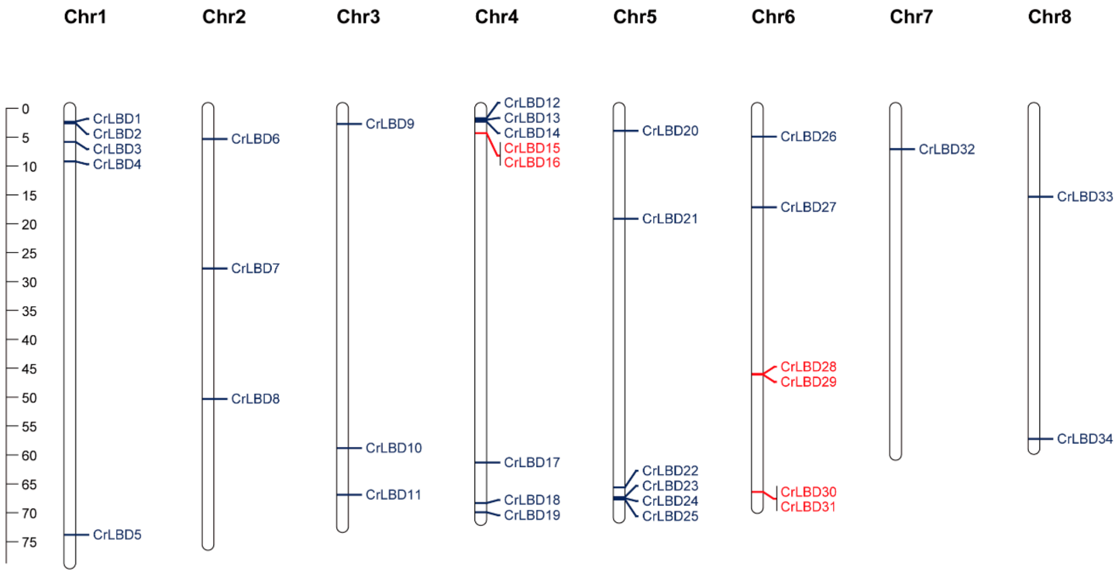

Figure S2. Chromosome distribution of 34 LBD gene family members in *C. roseus* (version 3).

Chromosomal mapping was based on the physical position of 8 *C. roseus* chromosomes. The scale on the left indicates the size of the chromosomes (Mb). The chromosome numbers are displayed at the top of each bar. The three pairs of genes are marked in red.

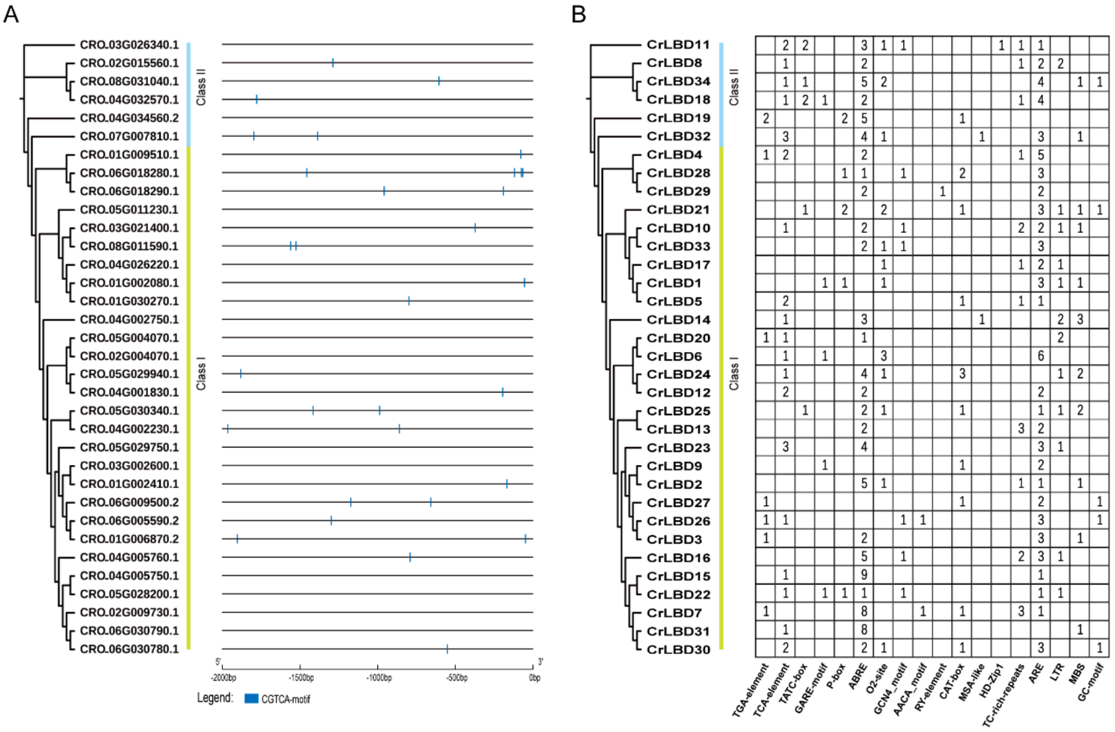

Figure S3. The *cis*-elements on putative promoters of CrLBD gene family members. (A) A phylogenetic tree of the CrLBD protein was constructed using the ML method and 1000 bootstraps. The green-yellow branches of the phylogenetic tree indicate LBD genes in class I; the blue branches indicate LBD genes in class II. The Schematic of the CrLBD gene family promoter with *cis*-element involved in MeJA responsiveness and the positions of potential elements are shown as blue bars. (B) We have compiled statistics on 18 *cis*-elements from three aspects: biotic or abiotic stress, plant hormones, and plant growth, as well as the distribution and number of these elements among various promoters of LBD gene family members. The phylogenetic trees in Figures A and B show the names and IDs of the LBD gene family members in corresponding positions.

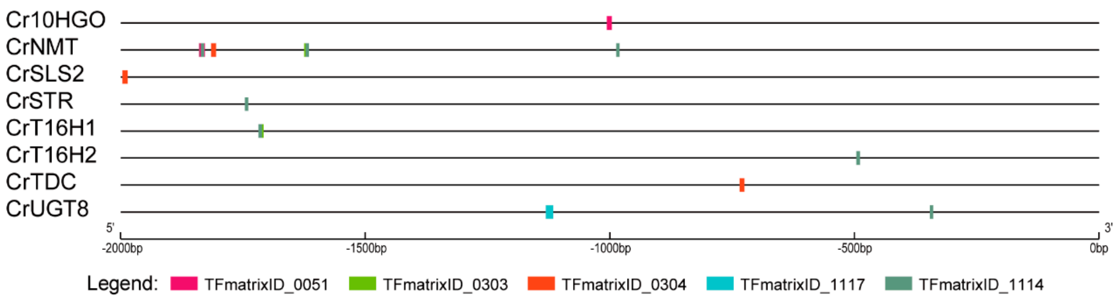

Figure S4. The *cis*-elements in the upstream of MIAs pathway genes in *C. roseus*. Schematic of the MIAs pathway genes promoter with *cis*-element involved in CrLBD responsiveness, the positions of potential elements are shown as colored bars. All potential *cis*-elements were predicted using the PlantPAN4.0 website.

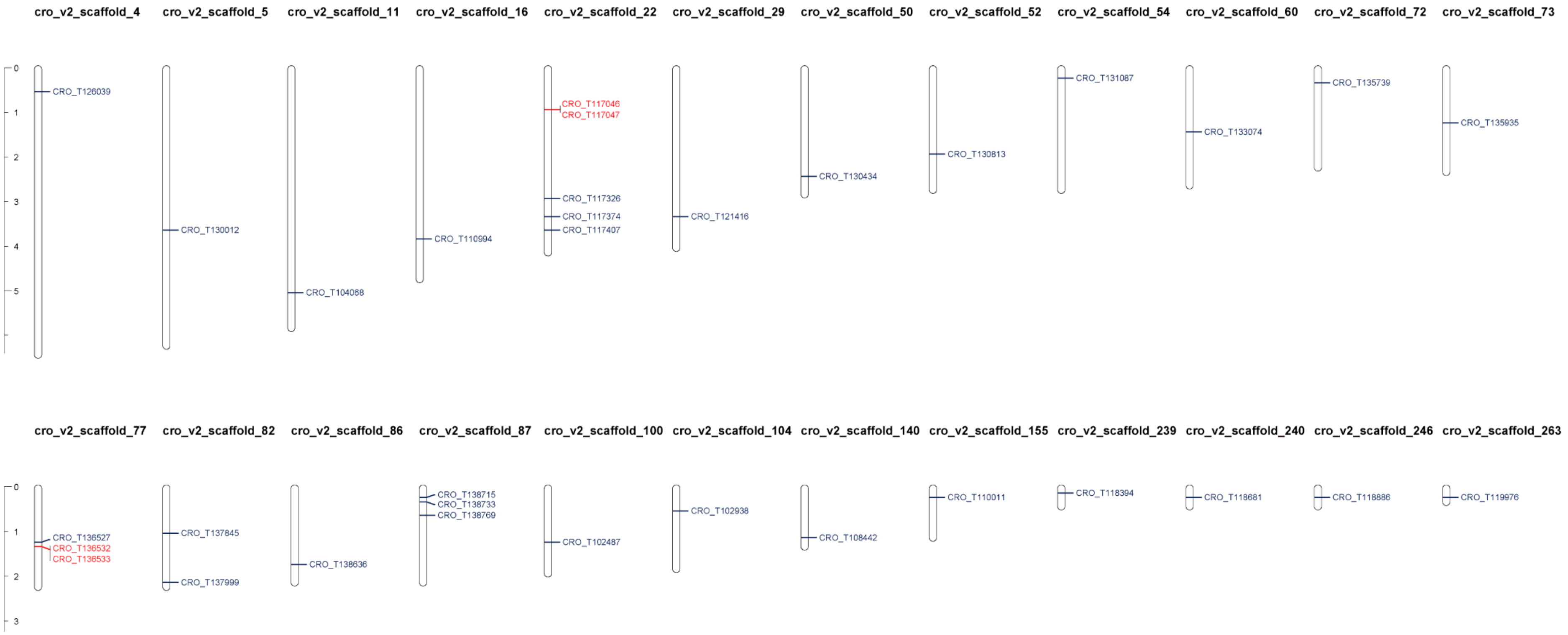

Figure S5. Chromosome distribution of LBD gene family members in *C. roseus* (version 2). Chromosomal mapping was based on the physical position in 24 *C. roseus* scaffolds.

The scale on the left is in megabases (Mb). The scaffold numbers are indicated at the top of each bar. The two pairs of genes are marked in red.
